# The Hospital Nursing Supplement

**Published:** 1894-04-28

**Authors:** 


### "The Hospital" Nursing Mirror.

Being the Extra Nursing Supplement of "The Hospital" Newspaper.

[Contributions for this Supplement should be addressed to the Editor, The Hospital, 428, Strand, London, W.C., and should have the word "Nursing" plainly written in left-hand top corner of the envelope.]

### News from the Nursing World.

### "THE HOSPITAL" CONVALESCENT FUND.

We are glad to be able to report that our readers have for some time now helped us to help others by contributing towards a "convalescent bed" in connection with an institution for the reception of weary workers recovering from illness, or in great need of rest and change. Useful as such a scheme must certainly be, some of our widely-scattered readers have pointed out that our plan of fixing on one spot had drawbacks. The journey was long for nurses in distant parts of the country, and the expenses of travelling proportionately large. The managers of the Fund are, therefore, arranging that in future nurses shall select their own locality, as far as possible, the Fund paying the whole or part of the expenses, according to the nature of the case. The most urgent will receive first consideration, and applicants must show that they are not able to secure what they need without the help of the Fund. They should address the Hon. Sec., The Hospital Convalescent Fund for Nurses, 428, Strand. Contributions should also be sent to the same address, and they will be acknowledged in the columns of the Nursing Supplement, where a short report of the progress of the Fund will appear from time to time. Our readers are reminded that this Fund is entirely dependent on their generosity and sympathy for its existence, and the extent of the help afforded to weary nurses is only limited by the amount of their donations. It is therefore hoped that subscribers will become more numerous, and that all will realise that time and trouble will not be grudged to promote so good an object as the one in question.

### USEFUL INFORMATION.

Surprise was expressed the other day by a medical officer of health at existing ignorance as to the facility with which an ambulance for the removal of an infectious person is to be obtained in London. It is highly probable that if this information were more widely known people would be less likely to break the law and imperil the safety of their fellow-creatures by surreptitiously taking the sick by ordinary vehicles. Every private and district nurse should be instructed that the Metropolitan Asylums Board will send an ambulance at any hour of the day or night to convey a person suffering from an infectious disease at the moderate charge of five shillings without, or seven and sixpence with, the services of a nurse.

### A BRILLIANT SUCCESS.

An admirably organised fancy bazaar in aid of the building fund of the Great Northern Central Hospital was held in the wards on the 18th, 19th, and 20th inst. His Royal Highness the Duke of York, president of the hospital, was there on the first day, when the Duchess of Teck, who had a stall of her own, formerly opened the bazaar. On Thursday the Baroness Burdett-Coutts was to have repeated the ceremony, but was prevented coming until later in the afternoon, and Her Royal Highness the Duchess of Albany graciously took her place, and on the following day the Duchess of Devonshire kindly consented to open the bazaar. Under such influential and kindly patronage it is little wonder that the undertaking has been a prosperous one. The fine circular ward and the long ward in which the stalls displayed their treasures were densely crowded each day. The officials are to be congratulated on the excellent management, which was conspicuous throughout, and on the exquisite taste of the floral and stall decorations. The latter held a famous variety of beautiful and useful articles, and the prices at which they were ticketted were so unusually reasonable that purchasers were visibly surprised at the good luck awaiting them. The substantial sum realised by the three days' sale is a testimony to the wide interest taken in the institution. The nurses' stall was deservedly popular, for the articles on it were as serviceable as they were cheap and tasteful.

### AN ENGLISH HEROINE.

Last week we referred to the heroic deed of an American nurse who displayed an unusual amount of physical strength and sufficient self-control to allow of her passing through a terrible experience in silence, rather than risk alarming an adjoining wardful of patients. This week we may dwell proudly on the heroism of an Englishwoman. A brave and loyal nurse, who, after working for nine long years under the Workhouse Nursing Association, was engaged some months ago at Newton Abbott Workhouse. Not unfrequently a resignation follows an appointment with suspicious rapidity, but Nurse Hinton did not beat a silent and hasty retreat, she stayed three months at her difficult post. She, as reported by the press, spoke openly of the existing evils, and she powerfully advocated the cause of the aged, the sick, and the imbecile. It is no light ordeal to face publicity as Nurse Hinton has done, although her sworn statement was, as the *British Medical Journal* well says, "but the culmination of a long series of charges which had been made against the management time after time by jury, by coroner, by a reforming minority among the guardians themselves, and by the press"—it is by a woman's moral courage that the exposure of grievous oppression has been effected. Nurses may well be proud of their sister's bravery, and will hear with pleasure that it has been proposed that a public recognition of Nurse Hinton's praiseworthy conduct shall take the substantial form of subscriptions to help to recoup her for the loss she has sustained whilst awaiting an inquiry, the result of which will prove of enormous value in advancing the cause of trained nursing in workhouses.

### ON THE RIVER.

A new steamboat has been added to the river ambulance service of the Metropolitan Asylums Board. It was launched on Saturday last, and contains ample accommodation for the removal of patients from the metropolitan area to the small-pox ships at Long Reach. There are rooms for the nurses, and also a special isolated place in which the friends of the patients can be conveyed.

### DISTRICT NURSING IN CARLISLE.

Good progress has been made by the Denton Holme District Nursing Association, which held its annual meeting lately. The ladies' committee appear to have proved themselves excellent administrators, and they report a balance in hand. They have been asked to consider the claims of the Botchergate District, and will gladly do so, we believe, if sufficient funds for the maintenance of an additional nurse can be collected.

### WISE MINERS.

The miners of Pelsall are wisely considering the requirements of their wives and families in the matter of good nursing, and they are said to be giving hearty support to a plan for securing it. The establishment of a thoroughly-trained district nurse is in contemplation, and it is to be hoped that this proposal will be unanimously supported in the neighbourhood of Pelsall.

### A VOLUNTEER WANTED.

Due appreciation appears to attend the work of the district nurses at Glasbury, and the hon. secretary was urged at the general meeting to try and obtain "the services of someone not entirely dependent on nursing for her livelihood, and who would live in Glasbury and be on the spot to attend any cases of illness that might occur, especially obstetric cases," reports the local press. A fully-trained nurse, with some means of her own, is doubtless the extra helper required, for certainly no imperfectly qualified person could undertake this important branch of nursing. Possibly some competent worker may be forthcoming, able and willing to give the desired assistance in caring for the sick poor in a charming part of the Welsh country.

### THE LATE MISS MARY GRENFELL.

The Mayor of Swansea recently convened a public meeting to consider the raising of a memorial to Miss Grenfell, and after much discussion a committee was formed to go into the matter. One speaker suggested the shape which the memorial should take might be postponed until the money was collected, and although this might simplify the matter temporarily, it hardly seems likely that the amount promised would be as large as if people knew the object in view. According to the local press the suggestions included almshouses, a district nurse, a new church tower, a memorial window, the endowment of a women's institute, a "miniature Exeter Hall for Swansea," the establishment of a ward at Swansea Hospital, and a contribution to the parish church restoration fund. Out of so many good objects it may be difficult to make a choice; but the hospital ward and the institution of a trained district nurse are probably the two works of practical utility which most strongly commend themselves to our readers.

### THE LANSDOWNE HOSPITAL.

In 1890 a visit was paid to Udaipur, in the Rajput States, by Lord Lansdowne, then Viceroy of India. The Maharana undertook to build a hospital in honour of the event, and a very fine building is the result of this determination. It has taken three years to erect, is entirely of stone, and is said to be a grand example of Rajput architecture. The front, which faces the street, is reported as most imposing, with two towers and some beautiful tracery. An operating-room, dispensary, receiving hall for patients, various wards, and the offices occupy the ground floor, whilst more wards, including some for paying patients, are on the first floor.

### BURGLARS AND NURSES.

The note which we gave last week on page xxii. under the heading of "Heroic Deeds" has called forth a private remonstrance from a cottage hospital matron, who declares that nurses are often very nervous at being left up alone—not of their patients, but of the possibility of the place being broken into. She asks us to fancy the mental agony a nervous person would suffer if she lived in anticipation of a burglar breaking in upon her. It was far from our wish to frighten nurses, and in case there be any who dread a visit from the burglar we will relate an experience which happened to us more than twenty years ago. A certain burglar was reported to have entered the hospital one night and to have made a careful inspection of its contents from end to end, including the residents' rooms. He found to his disgust that there was nothing worth taking away, and so returned empty handed to the street over the area railings. Pausing at the nearest lamp-post to see what o'clock it was, on feeling for his watch, he found that some one in the hospital had relieved him of it. English burglars have, not unnaturally, a poor opinion of hospitals, and nurses may feel reassured, seeing that such gentry are not likely to visit these institutions in the pursuit of their arduous calling.

### THE ROYAL BRITISH NURSES' ASSOCIATION.

### Three Resignations.

At the last quarterly meeting of the General Council it was decided to hold the annual meeting this year at Windsor, and to take new offices at No. 17, Old Cavendish Street. Dr. Bedford Fenwick stated that the reserve fund had been exhausted, owing to the heavy expenses in procuring the Royal Charter, and added that the audited accounts would show that the Association was still in a sound financial position. Dr. Bedford Fenwick resigned the office of Treasurer; Miss Grace Gordon that of Nurse Honorary Secretary, but she subsequently agreed to hold the post for the present; and Miss Daisy Robins resigned the office of Secretary and Registrar. Miss Daisy Robins is the third or fourth secretary that the Association has had, and she has done her work so well that she has given satisfaction to all the members. Her energetic work for the Association and her extreme kindness have been much appreciated, and no former secretary has been so helpful in attracting new members. The General Council very properly expressed the greatest regret at Miss Robins' resignation, and considered the loss of her services to be a matter of deep regret.

### Registered Nurses' Society.

If imitation be the sincerest flattery, then the Nurses' Co-operation, with its income of £20,000 per annum, should be gratified by the formation of this Society, which has been established by Mrs. Bedford Fenwick and is at present being worked from 20, Upper Wimpole Street. It is announced that the offices will be situated in Regent Street, and that Dr. Bedford Fenwick has assumed the office of Treasurer.

### On General Nursing.

By Rowland Humphreys, M.R.C.S., L.R.C.P.Lond.

### IX.—PNEUMONIA (*continued*).

### Stage of Red Hepatisation.

The characteristic sign of this stage is that of increased tactile vocal fremitus; in other words, the vibration of the voice is conveyed better to the finger when the lung is solid than in its normal condition. Another sign is that obtained by listening to the chest wall while the patient is speaking. The sound, again, is conveyed better than in health. Broncophony being the term applied to this condition. This sign is somewhat fallacious, because it depends on the bronchial tubes allowing the sound to travel down the air columns contained in them. At times the tubes become blocked with mucus, and then no sound is heard; sometimes the mucus shifts its position while the ear is listening, and then the conveyance of the sound is much improved. The breath sounds are also heard very distinctly, whereas at a little earlier stage they are very weak, the conducting properties of the inflamed lung for sound being diminished so long as there is little effusion and consolidation has not begun.

As the effusion increases and solidifies (as it soon does) the lung gives first a tympanitic or high-pitched somewhat resonant note, much as is produced when a wooden pencil or penholder is rapped lightly on the table, this is produced by the vibration of the air contained in the bronchial tubes embedded in the solid lung, and, probably, often assisted by the presence of a thin layer of uninflamed lung between the chest wall and the inflamed patch, *Bruit de pot fêlé*, or cracked pot sound, is the term applied to this peculiar sound.

As the consolidation of the lung advances, the sound becomes less and less tympanitic, and more and more dull and wooden-like. When quite solid up to the pleural surface, it gives a note similar to that produced by "percussing" the back of the hand. If the patch is deeply situated, there may be no perceptible dulness, and the other symptoms may be very obscure. The finger also experiences an unusual resistance to percussion. This is of course due to the increased fulness of the chest, or rather of the lung at the point under the spot which is being percussed.

On taking a deep breath the side of the chest affected moves less than the other side, and often it moves unequally in its different parts. The breathing itself is shallow and abrupt. The chest girth is increased from ½ to 1 inch on the affected side.

As to the expectoration, it is, as has been already remarked, thick, tenaceous, jelly-like at first. About the second day it becomes darker, and may become any shade of brown or rust colour, or it may even consist of bright blood in a more or less pure condition. The rusty appearance is due to the presence of blood in it, which has undergone more or less change. It is often preceded by a frothy expectoration from the bronchial tubes. The exudation into the alveoli is said to come from the smallest size bronchial tubes, which become stripped of their epithelium or lining at a very early stage. The expectoration proceeds from the same source. It is usually small in amount, and in many cases is entirely absent. When present, the rusty expectoration is characteristic of the disease, even when not accompanied by evidence of consolidation.

The stomach is upset, the tongue coated, and usually the bowels are confined. Frequently there is a breaking out about the lips, known as Herpes Labialis; and before the time when stethoscopes were first introduced it used to be considered one of the great symptoms of pneumonia. Now we recognise that it occurs in many other complaints, notably in quinsey.

The pain in the side which is often so troublesome is said to be due, not only to pleurisy, but to the irritation of the air passages, or to rupture of parts of the respiratory or other muscles, such as the rectus abdominis, from the violence of the cough.

In some cases vomiting is a marked symptom. In a case the author saw in consultation some years ago the vomiting had been so persistent that it completely hid the other symptoms of pneumonia, and the presence of lung trouble was only suspected from the blue appearance of the face. It should be remembered that although peritonitis, about which the mistake was made in this instance, is often followed by pneumonia; it is rarely preceded by it. As soon as the lung becomes solid from the coagulation of the exudation (which appears to be brought about by the action of certain bacilli) the air can no longer enter it, and the breath sounds cease to be heard; the lung becomes quite dull to percussion, the affected side of the chest becomes somewhat increased in size, even as much as one inch, and the stage of red hepatisation is now on the decline. In the course of a few hours the condition of "grey hepatisation" is reached, the lung presenting the same physical signs as in the last stage. The stages are not distinguishable in practice.

This consolidation takes place somewhere between the second and fifth day. The temperature is at its highest at about the beginning of this period, and it may rise to 107 even, and yet the patient may recover. It usually goes up a degree at night, falling again in the morning to a degree below the average. The temperature in pneumonia runs a very regular course. Within twelve hours or so of the first acute sign it runs up to its maximum, 104 or 105 Fahr., with a morning fall of two or three degrees and a similar evening rise. It keeps up till the crisis, which takes place between the fifth and eighth day. An additional rise takes place with the onset of any inflammatory complication, or if a further extension of the inflamed area takes place.

As already stated, the capilaries and, indeed, the vessels of the affected lung generally, are compressed by the exudation, and the flow through them impeded. The result of this is that the blood can neither get entirely rid of its waste material, which tends to accumulate, nor can it fully renew its supply of oxygen, while, more important still, there is a great strain thrown upon the right side of the heart, by far the weaker side. The heart has already suffered in its muscle from the fever, as in all cases of high temperature, and the additional strain often causes death from failure of that organ. Death rarely results directly from asphyxia, *i.e.*, suffocation; failure of the heart is the usual cause.

The inflammation is, at first, nearly always confined to one lung, and it very often attacks the base, or lower part of the lung behind. In three-quarters of the cases the right lung is affected, that is in adults. The difference between the two lungs is much less marked in childhood. If both lungs are attacked at the same time it usually means that there has been an old standing disease, which has weakened the lungs. A large area of the lung may suffer if the disease spreads from the original spot; a large area is not attacked in the first place. True acute pneumonia does not start in two places at once, thus differing from broncho-pneumonia. If the case goes on well it comes to an end by what is known as the crisis.

### Minor Appointments.

Greenock Hospital and Infirmary.—Miss Torrance, who was trained at the Paisley Hospital, and was afterwards Staff Nurse in Leith Hospital, has been made Assistant at Greenock.

### The Friends and Foes of Nurses.

### THE ROYAL NATIONAL PENSION FUND v. THE RECORD PRESS (LIM.)

### SOME PRESS OPINIONS.

#### LONDON DAILY PAPERS.

Daily Telegraph, April 16th, 1894.

It is satisfactory that the slur cast on a benevolent fund which aims at inducing nurses to lay by for a rainy day has been entirely wiped away. Nurses are exposed to many dangers and to much hard work, and it is especially important that they should not be discouraged from the prudent and really necessary plan of providing for the future.

Daily Chronicle, April 14th, 1894.

A case of considerable importance to nurses was decided this week in the Law Courts. There is an excellent institution known as the Royal National Pension Fund, supported partly by nurses and partly by wealthy persons who appreciate the services which nurses render to society. In no sense is it a trading concern, the welfare of the nurses being the only purpose that the National Pension Fund has to serve. How generally the Fund is appreciated may be gathered from the fact that the nurses contribute annually upwards of £30,000 in premiums. For some time past the *Nursing Record* has been giving currency to statements tending to discredit the *bonâ-fides* of the Fund. It is asserted that the premiums charged were from twenty to twenty-six per cent higher than those in force at old-established London insurance offices, and hinted pretty plainly that the motives of those who were conducting the Fund were not so disinterested as they were made out to be. The result, of course, has been to prevent many nurses from joining the association and deriving the benefits it offered, whilst a feeling of uneasiness has necessarily been created in the minds of those who were not in a position to verify for themselves the statements so positively put forward. When the case was brought into court it turned out that there was no defence, and no sort of attempt was made either to substantiate the categorical accusations of overcharging the hard-worked and poorly-paid nurses, or to justify the slur cast upon the National Fund. The Court was left to draw what conclusions it pleased as to the motives that actuated the conductors of the *Nursing Record* in disparaging an honourably-conducted and most useful fund. No one can tell how much harm may have been done to the interests of the nurses by the circulation of those statements; but the decision come to, by which an apology is to be published, in addition to the payment of damages and costs, ought to do something to repair the mischief, and to act as a warning to others to think twice before they exploit a noble profession, and traduce a helpful and indeed indispensable institution.

#### LONDON EVENING PAPERS.

Pall Mall Gazette, April 12th, 1894.

The Nurses' Pension Fund has just won an action for libel against the Record Press, which declared in some of its publications that very considerable premiums were its rule, and thus implied that it could scarcely be called a benevolent institution. Indeed the whole question was one of damages, for the defendants admitted that they were in error from the first. It is well that nurses should know that the Pension Fund does give them many advantages which they cannot obtain from ordinary institutions. We have looked through some of its reports, and find it an excellently conducted and in every way a commendable scheme for the assistance of women who never have any particular chance of saving money, but whose service to the State is none the less invaluable.

St. James's Gazette, April 12th, 1894.

In the interests of the nursing community we were glad to see that an end was made on Tuesday of the action brought by the Royal National Pension Fund for Nurses *v.* The Record Press (Limited). The defendants had published untrue statements to the effect that the National Pension Fund charged nurses much higher premiums than they would have to pay at old-established London offices. After a good many legal technicalities, the Divisional Court on Tuesday ordered the defendants to pay damages and costs and to publish extensive apologies, besides undergoing an injunction to restrain further publication. The Royal National Pension Fund has thus been relieved from a serious stigma; and the good faith of this very useful and well-administered enterprise is conclusively vindicated.

#### FINANCIAL PAPERS.

The Statist, April 21st, 1894.

A Libel Happily Squelched.—In our number for May 6th, 1893, we treated fully of the excellent objects and beneficial work of the Royal National Pension Fund for Nurses, and pointed out the inaccuracy and persistent ill-nature of the attacks made on the institution in certain quarters. The decision last week of the action for libel which the council brought in the Queen's Bench Division against the Record Press (Limited) renders it possible to speak of those attacks without reserve. It may be as well to state, first, the record of the Record Press Co. itself. The company was registered in September, 1891. Of the original seven subscribers six are solicitors, and the seventh a brother of one of them. The capital named is five hundred shares of £10 each. At the end of January, 1892, only 56 shares had been taken up (£560). Of these two lots of 25 each were held by the editor and solicitor of the company. A year later (February, 1893), 50 more shares appear to have been allotted to a benevolent maiden lady. The main object of the company was to acquire the business of the *Nursing Record*, a publication which presumably makes its profit out of nurses. The Royal National Pension Fund, on the other hand, is a benevolent institution founded, largely endowed, and carried on solely for the benefit of nurses.

The Nursing Record (Limited) took up a hostile attitude towards the Royal Pension Fund, and whether, as suggested by counsel, for the sake of 10 per cent. commission, or whether from pure "cussedness"—represented that the Fund charged nurses from 20 to 26 per cent. higher premiums than old-established London offices. At the same time they published a notice as follows: *Nursing Record* Fund. Annuity and sick pay given by two important English offices, *at ordinary commercial rates.* Application to the Editor, *Nursing Record.*

It might be thought that such an institution as the Royal Pension Fund could have treated the mis-statements of an insignificant opponent with the contempt which they deserved, but they would seem to form a part of a persistent and systematic attempt of certain people, who pose as friends, and somehow get the ear of nurses, to obstruct the kindly work of the Fund. The result of the action is as decisive as it is satisfactory. No attempt was made to justify the libel. It was a question of figures, about which there could not be two opinions. A fair critic would have compared his statement with the actual figures before making it, and acknowledged his mistake without being dragged into Court. Now the mis-statement has to be disowned and retracted by the following full and categorical apology [this public apology will be found in our advertising columns], which the defendant company is inserting in the *Times* and other papers.

We willingly give them a free insertion, and shall be glad to learn that every good nurse, and every friend of the Royal National Pension Fund, has had the gratification of seeing its terms.

Financial Times, April 13th, 1894.

It is a great pity, we think, that the course of legal procedure prevented this case from attaining greater publicity than it did. Everyone must sympathise with those who devote themselves to minister to the sick and suffering, and especially to the women-workers amongst them. It is abominable to find the efforts of philanthropists to alleviate the lot of such workers made the subject of libellous and unwarrantable innuendoes such as those for which an apology has now been tendered.

### "TRUTH" WOULD LIKE TO HEAR SOMETHING.

From Truth, April 26th, 1894.

I was asked the other day by a friend to say a word or two by way of assisting the Royal National Pension Fund for Nurses to repudiate the calumnious attack which formed the subject of an action recently disposed of. It seems to me, however, that this is like asking me to kill the slain. The daily papers contained a week or two ago a report of an appeal to a Divisional Court, which gave the public a pretty clear idea of the merits of the case. The *Nursing Record* published paragraphs stating that the National Pension Fund charged nurses "from 20 to 30 per cent. higher premiums than they would have to pay for similar advantages at old-established London offices." On libel proceedings being commenced, the proprietors of the *Nursing Record* found themselves utterly unable to justify their statement, and they sought to get out of the mess, and avoid a trial, by going before the Sheriff for the assessment of damages. The National Pension Fund, desiring that the matter should be threshed out in Court, contested this, and the dispute was eventually settled by an agreement for the publication of a retractation and an apology, and the payment by the defendants of 40s. damages and the costs of the action. I know that plaintiffs in libel actions are sometimes rather difficult to satisfy, but I really do not see what greater victory than this the managers of the Royal National Pension Fund could desire—always provided that the costs are paid in due course. The only further result that might have been attained by trying the action out is the elucidation of the circumstances under which such a baseless statement came to be put forward. On this, I confess, I should have liked to hear something.

*** Perhaps some of our many readers who know the true inwardness of the matter, and value the Pension Fund as a sure haven for nurses, will respond to the Editor of *Truth* by letting him "hear something" from them, at his office, Truth Buildings, Carteret Street, Queen Anne's Gate, S.W.

### A Trained Nurse for Droylsden.

Droylsden Nursing Association has recently become affiliated with the Queen's Jubilee Institute, and therefore the sick poor in that neighbourhood will in future possess what the Committee promises in its report, namely, "skilled nursing." We congratulate the Association on having secured the services of a thoroughly trained Queen's Nurse, and wish her a successful career.

### The Ethics of Nursing.

By Louis Vintras, M.B., B.Sc., Licentiate of the Royal College of Physicians, Member of the Royal College of Surgeons, Resident Medical Officer to the French Hospital.

II.

There is a proverb to the effect that one must swallow a certain quantity of dust in this life to gain admittance to heaven; it seems equally true that human beings are condemned to commit a certain number of faults. Faults will occur for some reason or another, and the wisest, like the humblest, experience the force of this law. However proficient a nurse may be, she is bound now and again to make a mistake, or to forget an order. A mistake is of double gravity when hidden, especially in medicine, because the physician or surgeon continues to act as if his former directions had been obeyed. To conceal a fault is an act of cowardice. By admitting an omission it may be rectified, by concealing it the wrong done may be irreparable.

"The more the merrier," is a familiar saying, but one which will hardly appeal to the average nurse when applied to the number of persons inhabiting a patient's house, especially in these days of excessive lecturing, when every young girl who has assisted at—I do not say followed—a course of white or red cross "first aids," thinks herself qualified, if not to undertake the duties, at least to censure the work of others. The nurse's position is a very delicate one. She will find many jealous eyes watching her, but if she has the confidence of the medical man who employs her, if she centres her thoughts entirely and solely on the patient, and mixes as little as possible with anyone in the house outside the sick room, she will greatly add to her own comfort, and tide over circumstances that would otherwise be unbearable. It must not be forgotten, however, that the friends of the sick person are sometimes irritable and apparently unjust from over anxiety; they often think that the physician and the nurse are hiding from them the true condition of the patient, and they become suspicious. The nurse should know her work thoroughly, but not assume to know too much about the case; many by a show of pseudo-knowledge, and by commentaries on the treatment adopted, though they may have enjoyed the temporary astonishment of a small circle, have wrought much harm and ruined their career. Medicine and nursing, though intimately linked, are scientifically distinct, and complete as her training may have been, proficient as she may be in all the details of her calling, the nurse must remember that of science she knows not, and cannot know as much as a first-year medical student. Bacon, in his twenty-sixth essay, says, "Seeming wise men may make shift to get opinion, but let no man choose them for employment"; and the same applies to women.

In the person the nurse is called upon to attend she must never see but a patient, whatever his faults, whatever his past career may have been, from the moment he is ill he becomes sacred to her as to all those around him. He suffers, and by her care she may lighten the burden of his sufferings; what more beautiful part can a woman play in this world. In the bearing of a nurse towards her charge there must be something of the indulgence of a mother for her child; that is why women are better nurses than men.

Nurses are recommended to be both gentle and firm in their dealings with their patients. It has often struck me that the equal cultivation of these two faculties must be a somewhat difficult task. Nurses, poetically speaking, may be angels, but in reality they are only mortals, and to expect to find in one being two such distinct and antagonistic qualities highly developed, is asking perhaps a little more than is just. As probably most nurses will find it necessary to choose between the two, I should strongly recommend the gentleness in preference to the firmness. It is astonishing what can be done with gentleness, especially when dispensed by a woman, and as the medical man is there, I think it would be as well if the so-called firmness, when needed, were left to him. She can always invoke the physician's orders for the refusal of any unreasonable request, and many patients are too often irritated by the exhibition of unnecessary coercion.

### The Nursing of Army Sisters.

By A. O. Grey.

II.

On arrival at Netley the junior sister will probably be sent to one of the divisions under a senior, who will initiate her into the manners and customs of military nursing. She will have the same privileges and allowances as the other sisters, and the same uniform, with the exception of the cape, which, until she has finished her six months' probation, and been approved by the Director-General, will be grey instead of the scarlet so proudly worn by the seniors.

She will in all probability be much surprised and a little taken aback when the strict economy in dressings and appliances is made plain to her. There are none of the elaborate antiseptics to which she has been accustomed and taught to consider essential. All wool, absorbent and otherwise, which has not been actually stained by discharge, must be carefully picked from the old dressing and made to do duty again. The cotton-wool used for wrapping up the limbs of rheumatics must be dried and reapplied, and the same system of economy must be observed throughout. The reason is a good one, but until experience teaches it is rarely realised. Government only allows a stated quantity, and if this is exhausted by what would be considered very ordinary use in a civil hospital, the supply runs short, and until the next issue of stores sad would be the condition of the sisters' dressing trays—instead of the nice rolls of wool only tow would be found. This, however, is not so apparent at Netley or head-quarter hospitals; at the smaller stations the supplies are usually far short of the requirements, dressings are issued by ounces, and very small ounces they are, too.

Again, the splints and instruments are not up to date, and in the operating theatre, when there is one, many of the modern conveniences are conspicuous by their absence. The great thing to learn is to make the most of everything, and by quickness of perception utilise such appliances as can be most readily procured. Training such as this is absolutely necessary to qualify a sister for active service where she would have a very limited supply of comforts, and even necessaries for the sick. No one realises how much can be done with small things until they are deprived of what in a civil hospital they would positively affirm were absolute necessities.

There will be a great deal that is strange to the new sister, even the rank of the patients will be puzzling perhaps at first, but it is wiser not to ask too many questions, observe much and say little. Soldiers are particularly alive to any discrepancy, and any remark out of which a joke may have its origin would serve to amuse Thomas Atkins long after the sister had forgotten the circumstance.

It will be found that a great deal can be done for the comfort of the patients; their confidence and respect gained, they are most loyal and grateful for anything that is done for them. When comrades from other wards meet, animated discussions may be overheard on the merits of their respective sisters. "Your's may be very nice, but she aint a patch on ours," and such remarks, show the appreciation of her service and cheerful help, which cannot fail to encourage her to be a friend to her men. Many poor fellows, coming home from India and other foreign stations, are in great need of sympathy and care, apart from their helpless physical condition. Frequently they have lost sight of their relations, and they are very thankful to the sister who listens kindly to their story, and who, if they are unable to write, will put them in communication with those from whom, by neglect or absorption in "fresh fields and pastures new," they have become estranged. Soldiers admire womanly dignity to what might be termed an abnormal degree, and an untold influence for good is in the power of the sister, who, in addition to skilled nursing, can give a higher tone to the wards of which she is in charge.

There are usually eight or ten sisters at Netley; of these, four are in charge of divisions, one in the sick officers' quarters, one on afternoon duty, and two on night duty. The probationers are assistants to the divisional sisters, or otherwise employed as the Lady Superintendent sees fit. Eight a.m. is the hour for duty, and the sisters are responsible for their divisions till the afternoon sister comes on. She carries out the directions left her in the books for the purpose, visits each ward occasionally, and bad cases frequently, does any urgent dressings, sees that the patients have tea comfortably, gives special nourishment, and has supervision over dysentery and enteric cases, so that no unauthorised food may be taken. When the size of the hospital and number of patients is considered, this will be acknowledged a most responsible duty. Though lasting only three hours, it is a most anxious time for the conscientious nurse. She reports any unusual circumstance, and makes a note of all medicines and stimulants given in the report books already referred to, before handing over the divisions to the regular sisters. The night sisters go on duty at half-past eight, and carry out all the instructions left them.

During the trooping season the entire length of the hospital (over a quarter of a mile) is required to accommodate the sick, and many cases requiring the utmost care are brought by the ambulance, when the special trains bring the invalids from Portsmouth, where they disembark. Some of the poor fellows are so emaciated that the wonder is they live to reach the wards. Malarial fevers of all kinds, and dysentery, such as is never seen in civil hospitals, require ceaseless care and nursing. Only people who have never been in a military hospital, and are totally ignorant of the lives wrecked by climate and disease, could speak lightly of the work and responsibility of an Army Sister.

### Hospital Treatment.

Some readers may have noticed a letter signed "Alice Court" which appeared under this heading in the *Echo*. It contains most heart-rending details of the alleged inhuman treatment of the writer, who was first admitted to King's College Hospital and then sent adrift in a fainting condition "in pain, hungry, and almost dead from tiredness."

We have communicated with the authorities of King's College Hospital, and have been informed by them that "'Alice Court' is not the writer's real name, nor is "it a name assumed merely for the purpose of writing "to the newspapers. She entered the hospital under "that name, and when asked why she had given a wrong "name her reply was that none of the hospitals would admit "her if she gave her correct name. She is a Miss Beatty, who "lately brought an action against an eminent physician on the "staff of St. Thomas's Hospital, to get damages, alleging that he "had performed an operation without her consent. Miss Beatty "lost her action, the verdict being given for the defendant, "as reported in the newspapers about a week ago. Miss "Beatty was admitted into King's College Hospital, under the "name of 'Alice Court,' as a governess—apparently she was "a nurse—on Saturday, March 17th, and discharged on the "following Wednesday. In her letter to the *Echo*, signed "'Alice Court,' she complains of being discharged from the "hospital. Why she was discharged, she says, remains to be "answered. The answer of the hospital authorities is 'that she "was quite well enough to go out.' It appears that though "discharged on Wednesday she was allowed to remain until "the next day, but early on Thursday morning, before six "o'clock, she determined to go out. She returned, however, at "half-past nine a.m., asking to be readmitted. This was refused, and as she would not leave the hospital, it was thought "best to send her away in the care of a police constable, it "being explained to him that there was no charge against the "lady—only she would not leave the hospital." Such are the material facts, as stated by King's College Hospital, and they do not seem to call for comment.

### Women's Work.

### HOW TO BECOME A COOKERY TEACHER.

The recent demand for technical instruction has opened out new branches of employment for women with practical and domestic tastes. The domestic arts have suffered a long period of neglect during the great struggle for women's intellectual enfranchisement which has specially characterized the Victorian era. So anxious have its promotors been to gain for our girls the same opportunities of mental training enjoyed by their brothers, that the domestic training which our grandmothers underwent has been utterly neglected. The simple homely occupations of cooking, clear starching, and sewing have been looked on as almost beneath contempt. Nay, they seemed in danger of becoming lost arts, servants taking their cue from their mistress with such success that a good plain cook, a decent laundress or sempstress were advertised for in vain.

It would be difficult to over-estimate the resultant discomforts and drawbacks and pecuniary losses, but happily the evil has wrought its own cure, and greatly to the general well-being the pendulum is on the backward swing. Modern technical instruction for women includes in its code, cookery, laundry work, sewing, and dress-making, and in every county there is a demand for teachers of all these branches. Cookery is the best paid of the three, but many teachers qualify in one or both of the other branches as it is a gain to themselves and to their employers if they can hold classes in more than one of the subjects.

No one should go in for the training in cookery who has not fairly good health and enough education to pass the easy examinations in physiology, hygiene, and chemistry. To make a successful demonstrator the student must also be possessed of a good delivery and the power of imparting knowledge in a pleasant and interesting way.

### Choice of a School.

The next point to be considered is the choice of a training school, which should be one of those whose diplomas are recognised by the Education Department, otherwise the student would not be qualified to teach in Board schools. The two principal and original training schools are those of Liverpool and the former South Kensington one, now moved to Buckingham Palace Road. Would-be students can get all particulars by addressing themselves to Miss Lucas, Liverpool Training School of Cookery, Colquit Street, Liverpool, or to the Secretary, National Training School of Cookery, Buckingham Palace Road, London, S.W. Both schools have been established some years, and have done good work. The committee of the Liverpool School have from the first paid special attention to the teaching of artisan cookery. Many branch schools have been started in different counties all over England, with teachers trained at Liverpool, where pupils who have passed the practical and theoretical examination can obtain a teacher's diploma from the Liverpool and National Union for Technical Education of Women in Domestic Sciences. There is also a school at Glasgow in connection with this Union. The training for the full diploma in all branches of cookery occupies at least nine months, and costs £14 14s., examination and diploma fees and cost of books amounting in addition to about £1 10s. Beyond a few books and a supply of aprons and kitchen cloths no other "stock-in-trade" is requisite for the student. The fees for an artisan diploma, which will qualify a student for teaching in all Board Schools, costs £9 9s.

In the National Training School of Cookery the fee for a teacher's diploma in plain cookery, equivalent to the artisan diploma, is £13 13s., the training lasting for six months. After qualifying in the plain course, the student must train for five months more and pay a further fee of £21 in order to obtain a teacher's full diploma. There is no doubt that under this syllabus students get a more thorough training in high-class cookery, which may certainly be useful to those who wish to take advantage of such openings for work, as, for example, going out by the day to cook for dinner parties—work which a good many ladies are taking up now in London, and which is well paid. It must, however, be borne in mind that in none of these schools is this training intended to turn out a simple cook, but a teacher of cookery; that is, a person well instructed in the principles—chemical, economic, and physiological—of cookery, and able to illustrate it by practical experiments, viz., in the preparation of certain dishes. Unless the would-be teacher has had a certain amount of experience in cooking, or is specially apt, or has had opportunities of supplementing her training by practising at home, the nine months' training will not fit her to enter into the lists against a professed cook of many years' standing. As a matter of fact, probably 50 per cent. of young and newly-fledged teachers have hitherto purchased their experience at the expense of their classes, which is most likely the reason why Liverpool has lately made nine months, instead of six months, the minimum time admissible for the training.

If the student has passed with credit and gained the full diploma she will probably have little difficulty in obtaining work under one of the County Councils. She will, perhaps, think herself most lucky if she be appointed to a permanent post in some town as a centre, where her work will consist in teaching in the elementary schools and holding artisan and household classes both in the town itself and in the immediate neighbourhood. In the country her district may be much larger, and her work will be that of an itinerant teacher, going from one out-of-the-way village to another, and perhaps having to take about with her a portable stove and a hamper of utensils. A teacher is not supposed to give more than ten lessons of two hours each in the week. This sounds less than it really is, when travelling is added, and the half-hour or more of preparation such as weighing out materials and putting things ready generally for a class. The room, fire, &c., is always supposed to be ready, and a woman ought to be in attendance who can act as kitchen maid to wash up, pack, and unpack the hampers, but if the local committee is incompetent, the teacher's patience may be much tried by finding confusion instead of order, the fire unlit, and suchlike *contretemps*. Salaries average £80 for those holding full diplomas; temporary engagements are usually paid for at the rate of £2 2s. a week, and travelling expenses in all cases. If the teacher can give classes in some other subject in the technical line or can lecture on nursing she could easily make £100 to £120 a year.

In conclusion, the writer can state emphatically from her own experience that she does not think anyone will have cause to regret the time and money spent on the training. Even if any student should find herself at the end of the course unequal to demonstrating, she may still become a staff teacher in a school, or be well qualified for the post of matron in a home, &c. We hope the time may not be far distant when every English girl will feel that a knowledge of cooking is as necessary a part of her education as the three R's, and the numerous and excellent classes now started all over the country put it within the means of everyone to quality herself for becoming a good housekeeper and mistress of her own house. In this case, as in everything else, knowledge is power, and the mistress who knows something of cooking will no longer be at the mercy of a careless or dishonest cook. There is no nation so wasteful and ignorant in their methods of cookery as the English. Any French, German, or Italian family can live far better on half the wage of an English artisan, simply because they have not neglected this useful art. No doubt our very prosperity has helped to cause this, but if recent "bad times" should have the effect of bringing about a remedy then indeed we shall see how good may often spring out of evil. E. L.

### The Great Northern Central Hospital.

Whether in its festival dress of last week when red cloth and stands of flowers, bands of music and entertainments, many and various, betokened the presence of Royal and loyal guests, or in its ordinary work-a-day aspect the Great Northern Central Hospital in Holloway Road is both attractive and interesting. Structurally it possesses many of the most modern sanitary improvements, and the tiled bathrooms and ward pantries, the marble slabs and polished floors, all point to the continued crusade against dust and dirt of all kinds, which science and taste alike now dictate. Every visitor to a circular ward, and there are two at this hospital, is struck at first by its obvious advantages, and it is only perhaps to a nurse's mind that one definite drawback to this form of architecture presents itself. Wherever she stands some beds are hidden by the central support where the fireplace or stove is located in the middle of the room. Now a good nurse likes to have all her patients within sight, especially when on night duty. But even a straight ward does not always enable her to secure this important end.

At the Great Northern open verandahs form pleasant airy spots for convalescent patients, and the central position chosen for the Matron's room is excellent, whilst the new rooms for nurses, private patients, &c., leave nothing to be desired. With regard to the small private rooms we can imagine no greater boon to a sick person of moderate means than to be able to secure skilled treatment and nursing allied to the privacy to which social his position has accustomed him. Whilst the terms asked will probably not be prohibitive, they should be such as to cover the cost which will be entailed on the institution by this new department. Private patients admitted to the Great Northern Hospital may well be congratulated on the comfort they will enjoy, for it is obvious that the sick are of the first consideration there. This was well shown whilst the bazaar was in progress, for the inhabited wards were absolutely isolated during those three days. No detail of the ordinary routine was interfered with, and, save when the Royal visitors went quietly round talking to the sick folks, nothing occurred to show that unwonted events were taking place in the building. With constant evidence of the excellent work of the Great Northern Central Hospital, and of the kindly spirit which prevails in that much-needed institution, we hope that poverty will not be allowed to cripple in any way its usefulness.

### The Hospital for Sick Children.

A correspondence has been published by *Truth* in which the Matron who acted as *locum tenens* at this hospital informed a candidate for a probationer nurse that if she was a Unitarian she could not be received for training. Immediately this came to the knowledge of the committee they repudiated the action of their temporary officer in setting up without their knowledge or authority such a thing as a religious test in an absolutely unsectarian hospital, where no questions are allowed to be asked about religion, nor is any candidate to be rejected on account of religion. From inquiries we have made, we have ascertained that the unsectarian character of this hospital is proved by the fact that there are Roman Catholic and Unitarian nurses at present on the staff, and that some of the Matrons of the Home at Highgate have been Nonconformists. If there is one fact more true than another about English hospitals it is that they are admirably free from bigotry, and that religious tests are never imposed upon the patients or the staff, all being free to receive the ministrations of the ministers of their own denomination. We offer the committee of the Hospital for Sick Children the expression of our sympathy, and are glad to note that *Truth*, with commendable fairness, does its best this week to free the hospital from any taint of sectarianism, of which in reality it has not a trace.

### The Late Mrs. Wardroper.

The beautiful memorial which has been erected in St. Thomas's Hospital Chapel to the memory of the late Mrs. Wardroper is to be unveiled by the Archbishop of Canterbury on Monday, April 30th, at half-past four p.m. No worker in the great nursing cause could be more deserving of such a token of affectionate remembrance than Mrs. Wardroper, who rendered such inestimable service to the great hospital of which she was Matron for thirty-three years, and to the whole of the hospitals of the world. Miss Nightingale truly speaks of her as

"A perfect woman, nobly planned
To warn, to *comfort* and command."

"'Comfort,' not in the present meaning of comfortable, easy-chair life, but 'comfort' in the good old meaning of 'Be strong with me.'"

The memorial in St. Thomas's Hospital Chapel bears the following inscription:—

To the memory of Sarah Elizabeth Wardroper, a faithful servant of God and man; the working leader in a great reform, quietly and peaceably pursued, by which the care of the sick took its right place as a high and holy calling, that enlists the noblest qualities of heart and mind, and turns to efficient use the intelligence, refinement, and devotion of good women. For 33 years Mrs. Wardroper rendered faithful service as Matron to this Hospital. Selected by Florence Nightingale as the first Superintendent of the School of Nurses established by her in this Hospital, she was successful during the last 27 years in training and sending out into our own and other lands capable women, worthy to carry on the good work. She retired from her post in June, 1887, and died at East Grinstead on the 14th day of December, 1892, in the 80th year of her age.

"I was sick and ye visited me." "Inasmuch as ye have done it unto one of the least of these my brethren, ye have done it unto me." Matt. xxv. chap., v. xxxvi. and xl.

This Memorial has been erected by the Governors of this Hospital and others who knew and appreciated her great qualities.

### Appointments.

Ayr County Hospital.—Miss Tod, Night Superintendent at the Western Infirmary, Glasgow, has been appointed Matron at the Ayr County Hospital, and we wish her every success.

Weston-super-Mare Children's Home.—Miss Caroline Fishwick has been appointed Matron of this Home, which is in connection with the Bristol Children's Hospital. She was trained at the London Hospital, and was afterwards Staff Nurse there; Nurse at the West Norfolk and Lynn Hospital, Night Superintendent at the Bristol General Hospital, where she took holiday duty as Assistant Matron. We congratulate Miss Fishwick on her appointment.

Victoria Hospital for Children, Chelsea.—Miss Hamilton has been appointed Matron of this hospital. She was trained in the Nightingale School, St. Thomas's Hospital, and held the post of Assistant Matron there. We congratulate Miss Hamilton on her appointment to this charming children's hospital, and also the committee in having secured a fully-trained and experienced hospital worker for so important a post.

Taunton and Somerset Hospital.—Miss S. Harriett Harris has been made Lady Superintendent of this hospital. She was trained at the London Hospital, where she was afterwards Staff Nurse. Miss Harris has done private nursing in the South of France in connection with the Hollond Institute. She has also lectured on nursing with marked success in country districts. For the last eleven months she has been a Sister at the Taunton and Somerset Hospital, therefore the committee have personal knowledge of the abilities of the lady whom they have selected as successor to Miss Marion Wilson, who has achieved such a marked improvement in their nursing school. We wish Miss Harris every success in her new new and responsible position.

☞ For Everybody's Opinion, Reading to the Sick, Book World for Women and Nurses, &c., see page xxxix. et seq.

### Everybody's Opinion.

TO CORRESPONDENTS.—We would remind correspondents once again that no notice can be taken of any communication which is not accompanied by the name and address of the writer. We shall be glad to have the name and address of "An Ordinary Male Volunteer," and also of "An Orderly."

### THE ROYAL NATIONAL PENSION FUND FOR NURSES.

THE Council of this Fund have been reluctantly compelled to bring an action against the publishers of statements to the effect that it charged nurses premiums exceeding by from 20 to 26 per cent. those they would have to pay for similar advantages at old-established London offices. The Council ask you, therefore, to allow them to state for the information of nurses and the authorities of hospitals and kindred institutions throughout the British Empire that the action resulted last week in a judgment in favour of the Fund for 40s. damages in addition to the costs, an injunction restraining the defendants from further publishing the statements, and a public apology which was advertised in the *Times* of April 19th, 1894, and subsequent days. The action having been brought, in the words of Mr. Finlay, Q.C., to counteract the mischief "that the publication of the libels was intended to do," the plaintiffs waived their claim to substantial damages. The investments of the Royal National Pension Fund now exceeds £175,000, including a Donation Bonus Fund of upwards of £40,000. There is also a Benevolent Fund of more than £11,000 for the benefit of those whose means of saving have proved insufficient to secure for them an adequate provision against sickness or incapacity, however caused, and for old age. The Donation Bonus Fund and the Benevolent Fund, combined with the principle of mutual insurance, honorary management, and great economy of administration, enable the Royal National Pension Fund to offer to all workers among the sick throughout the British Empire substantial and exceptional advantages which the Council believe to be not otherwise procurable. The Council are anxious to let nurses in every part of the world know that the misleading statements, now admitted to have been without foundation or justification, in respect of which the action was brought, and which have been very widely circulated amongst them, have been withdrawn and altogether disavowed. For these reasons it is hoped that the Indian and Colonial papers will republish this letter for the information and protection of nurses working in every portion of the empire.

Signed on behalf of the Royal National Pension Fund for Nurses.

(By order) LOUIS H. M. DICK, Secretary.

8, King Street, Cheapside, E.C.

### ETHICS OF NURSING.

"EX-NURSE" writes: I think it a great pity that Dr. Vintras should, in his paper on the Ethics of Nursing (see "Nursing Mirror," April 14th), make the following statement, "A patient is an inferior being," &c. This, I think, is a very dangerous doctrine, and one that only too many nurses are ready to carry out. Taken with its context, I consider the nurse who worked with this point of view in her mind would be little likely to awaken "confidence" in her patient. Many of the difficulties that at present arise in private nursing are due to the fact that nurses are only too ready to treat all their patients as though they were children, neurotics, or idiots. It is quite natural that a large number of patients who, though weak, are in perfect possession of their faculties, should strongly object to this form of treatment. I think that a more rational view is a needful one to encourage, and that nurses should remember that patients, though ill in body, are often clear in mind, and are perfectly capable of criticising with slightly amused annoyance the brief authority in which it pleases their "ministering angel" to clothe herself.

### For Reading to the Sick.

### LIFE'S TRIALS.

**Motto.**

Learn as if you were to live for ever;
Live as if you were to die to-morrow. —*Proverb.*

**Verses.**

The path of sorrow, and that path alone,
Leads to the land where sorrow is unknown.
—*Cowper.*

We did amiss when we did wish it gone
And over; sorrows humanise our race;
Tears are the showers that fertilise this world,
And memory of things precious keepeth warm
The heart that once did hold them. —*J. Ingelow.*

Self love, no grace in sorrow sees,
Consults her own peculiar case;
'Tis all the bliss she knows;
But nobler aims true love employ;
In self-denial is her joy,
In suffering her repose! —*Cowper.*

**Reading.**

Before I was afflicted I went astray, but
Now have I kept Thy word. —*Psalm*, cxix. 67.

"Elijah went a day's journey into the wilderness, and came and sat down under a juniper tree, and requested for himself that he might die, and said, 'It is enough now, O Lord, take away my life, for I am not better than my fathers.'" . . . Who amongst us has not, in times of depression, felt an echo in his own soul of Elijah's cry, "O Lord, take away my life?" I think it was no one great trouble, but rather an accumulation of small trials and discomforts, that made the prophet break down so utterly. It is true that Jezebel had threatened his life, but that was no new thing to him. Physical causes seem to have had much to do with his state at this time. He had left his faithful ministering servant behind him, and had been journeying all day under a hot sun across a sandy desert. And now, towards evening, weary, hungry, thirsty, he threw himself down under a juniper tree. What wonder that his spirit was cast down within him! What wonder that he took a gloomy view of his spiritual progress, and felt that, notwithstanding all God's revelations to him, he was no better than his fathers!

Was the Lord angry with His child because, being thus wearied out, his spiritual life seemed ebbing away? He spake no word of anger. The prophet was weary, and so God gave His beloved sleep; he was hungry, and so bread was provided for him; he was thirsty, and so a cruse of water was placed at his head. Thus, without chiding, his heavenly Father supplied his wants, and thereupon Elijah rose up in fresh strength to do his Master's work.

Are you lying footsore and weary under your juniper tree, feeling as if God were dealing hardly with you? Do you find the daily cares and worries and perplexities of life almost more than you can bear? Take courage! Our God is a God that changeth not. As He gave rest to the weary prophet, so, if you bring your troubles to Him, you shall find rest for your soul. Your hungering heart will meet its satisfying portion in His love, and your thirsty spirit will drink of the fountain of life, and so be satisfied; and thus you also shall be strengthened, and go on your way rejoicing, willing to do the Master's work in the Master's way. C. M.

Unloving words are meant to make us gentle, and delays teach patience, and care teaches faith, and press of business makes us look out for minutes to give to God, and disappointment is a special messenger to summon our thoughts to heaven. E. M. SEWELL.

### The Muses' Looking Glass.

WANDERINGS IN ITALY—A GENOESE CEMETERY.

The old Protestant cemetery at Genoa stands on a hill outside the city walls, and from it one of the most glorious views can be obtained of distant purple hills and the snow-covered Maritime Alps, with the blue Mediterranean as foreground.

The rocky hill, on the summit of which the cemetery stands, has just sufficient soil for the usual cypresses and small plants, but the graves themselves have to be excavated in the solid rock; soil is precious round Genoa, and this was the only spot which the authorities in the old days would grant as a last resting place for the stranger heretics who died among them, and a very beautiful little God's acre it is; but unfortunately it is full, and now foreigners have to be buried in a special plot assigned to them in the huge Campo Santo at Staglieno.

This Campo Santo is considered as one of the sights for strangers, the natives being intensely proud of it as their modern sculpture gallery. It consists of a series of cloisters, and people are buried in different parts according to the price they pay. The second-class graves are merely shelves one above another, partitioned off at intervals of six feet, each division when occupied by a coffin being slabbed over and inscribed.

The first-class graves, however, form the chief sight along which the weary traveller is expected to wander, and at each step all his cherished traditions of Italy as the nation of artists receive a fresh shock. These "graves" consist of huge white marble erections representing family scenes with life-size sculptured figures, a favourite being the death-bed of the person in whose honour the monument is raised. His wife and relatives stand or kneel round the bed in studied attitudes and with conventionally simpering expressions, and all are costumed and *coiffé* in the latest mode, everything they wear being most faithfully reproduced, from the pattern of the tweed on the son's coat to the handle of his umbrella, which, with the latest edition of the Caffaro stuck under his arm, and his hat, which he has somewhat unnecessarily introduced into the sick room. The women of the family have specially grand gowns made on purpose to be sculptured in, the trimming and texture being carefully modelled, the skirts, in some instances, drawn up in a careless fashion to show a lace or embroidered petticoat below. The ladies always have their hair done by a noted coiffeur for the occasion, and sitting as models for these ghastly images takes up many months of their time.

The likenesses are generally excellent, and the originals are often seen standing contemplating their counterpart presentments with keen enjoyment. Sometimes the scene is laid on the terrace of the dead man's house, where his family gather round, some standing and others sitting in more or less graceful attitudes, the women-folk busy with a piece of fancy work, or else engaged in a lively *tête-à-tête* with presumably a brother-in-law, everybody of course being habited in the latest fashion.

A Genoese girl said she had had a gown from Worth for the occasion, and her delight was great at the effective way in which all the little frills upon it were shown up in the marble. The dead person is usually an unobtrusive detail in the composition.

For these monuments people contentedly pay thousands of francs, a widow frequently being reduced to semi-starvation in order to put up a sufficiently grand one to her husband's memory.

One old lady related how she had had herself sculptured in her Sunday clothes ready to adorn her own tomb, and it really had a very chilling effect to see her standing exactly in "her habit as she lived" petrified into white marble, all her clothing being reproduced with abject fidelity, even to the elastic-sided boots, with the elastic slightly worn, and the kid gloves with little flaps at the ends of the fingers (her gloves always were a few sizes too large).

Mazzini's tomb is hewn in the solid rock in the hill side, an especial honour, but it is too much of an ascent for any but his most devoted admirers to attempt. The latter may occasionally be seen laboriously climbing up in a long row, each carrying a wreath of either paper or tin flowers to lay near his grave. This sight is usually observed when the Government has been doing something vexatious, and republican spirits find this a convenient method of expressing their sentiments, for it has the charm of a protest without the danger, and at the same time a pleasant picnic is enjoyed.

The only Christian part in this oppressive sculpture gallery is the part devoted to the poor; they are buried in the ground in the centre of the quadrangle, and their graves are marked by simple wooden crosses. All feelings of peace and fitness are rudely dispelled on hearing that this resting place is theirs for only five years, after which time they are dug up to make room for others in the precious ground, their bones being all put together in a deep pit or else burnt.

Kossuth's wife and daughter were among the many foreigners to whom Genoa first offered a resting place and then a tomb.

### Where to Go.

Society of Lady Artists, Drawing-Room Gallery, Egyptian Hall, Piccadilly.

The private view of this society's works was held on Saturday. By no means a small exhibition, a standard of general excellence was maintained throughout. Many, indeed, of the drawings we found exhibited here on Saturday could fairly stand a severer test than being judged by the standard which is ordinarily applied to "woman's" work. In this particular instance a more crucial test could be applied, one not restricted to sex or nationality. Take for example the works of Madame Louisa Starr Canziani, one of the professional members of the society. Especially in the case of her portrait of Mrs. Wigan (No. 295), the artist displays herself not only as mistress of techique, but as an original worker and thinker. But other names stand out conspicuously among the 394 pictures which adorn the walls of the Dudley Gallery. Miss Ida Lovering in her study (No. 191), "A Chase," shows herself at her best; this is a charming subject, equally charmingly treated, simple, fresh, and suggestive. Miss Fairman is represented in some clever studies of animal life and also in landscape work. A large oil (No. 47), "Spring" (by L. Edwardes-Jones) is deserving of better hanging—but sky-high, yet attracts our notice in a forcible manner. The society may fairly congratulate themselves on the attractiveness of the present exhibition.

### Notes and Queries.

### Queries.

(48) *Chiropody.*—Recommendation of teachers wanted.—*R. T.*

(49) *The Continent.*—Addresses of Nursing Institutes wanted.—*Nurse B.*

(50) *Training.*—Will you tell me if there is any chance of my getting into a children's hospital at eighteen?—*D. V. C.*

(51) *Work.*—Can you advise me as to work in workhouses?—*E. K.*

(52) *Address Wanted.*—Will you kindly tell me the address of the solicitors in the R.N.P.F. case?—*Nurse A. P.*

### Answers.

(48) *Chiropody* (*R. T.*)—We cannot undertake to recommend special teachers.

(49) *The Continent* (*Nurse B.*)—See Burdett's "Hospital Annual."

(50) *Training* (*D. V. C.*)—You are too young to begin hospital work. See "How to Become a Nurse," by Honnor Morten, published at 428, Strand.

(51) *Work* (*E. K.*).—Write to Hon. Secretary, Miss Lee, 20, Bryanston Street, London, W., for information about workhouse attendants.

(52) *Address Wanted* (*Nurse A. P.*).—Messrs. Baker and Nairne, 3, Crosby Square, E.C.

*Mrs. Heriot-Hill* has received so many answers to her advertisement for a trained midwife that she regrets her inability to reply to them all.

### The Book World for Women and Nurses.

[We invite Correspondence, Criticism, Enquiries, and Notes on Books likely to interest Women and Nurses. Address, Editor, The Hospital (Nurses' Book World), 428, Strand, W.C.]

### The Yellowbook.

The long-expected "Yellowbook" has come at last! The press had worked up our enthusiastic expectation, and its entry into literary London resembled that of some foreign Shah or potentate, from whom everything strange, fascinating, and bizarre might be expected. Curiosity is now assuaged, or may easily become so at an outlay of 5s. And what do we all think now that we have read it and scanned its pictures? Firstly, we think the idea of publishing a nice fat book of short stories, by the very best talent available, and giving them a durable linen cover, was a very happy thought. We love our little "Pseudonym Library," which contains some of the best short stories ever written, but one Pseudonym will not pass a railway journey of greater than suburban length, and even in its paper cover it costs 1s. 6d. We cannot buy three linen-covered Pseudonyms for 5s. The "Yellowbook" gives us five stories (one by Henry James is the best he ever wrote), poems by the best among the new men (Le Gallienne, A. C. Benson, Davidson, Gosse, &c.), two plays, one of which is the joint production of John Oliver Hobbes and George Moore, and four clever essays. Were this not generous measure, even without the illustrations? Sir Frederick Leighton contributes two beautiful classical studies. Aubrey Beardsley's portraits of Mrs. Patrick Campbell are amazingly like, they have caught the spirit of the original in a magic way; one marvels at the dainty grace of line and the bold use of masses of black shadow. For these alone the Yellowbook is worth more than its price. Walter Sickert's "Old Oxford" is a masterly rendering of a most difficult subject. The cross lights are enough to have bewildered any painter of lesser resource and scientific knowledge, but he has treated them with evident facility and triumph. Joseph Pennell's "Puy en Velay" is a piece of pure Dürer, quaint and interesting, as well as intrinsically beautiful. Mr. Rothenstein's work is as clever as we have learnt to expect from him, but can scarcely be called interesting. Mr. Furse is represented by a reproduction of his portrait of a lady, now being exhibited in the New English Art Club. Nettleship and Housman contribute rather weird drawings, such as are more admired by the modern Belgian than the up-to-date Englisher; and Anning Bell, a book plate for General Gosse, which strikes one as singularly inappropriate for a man of war. To return in rather more detail to the literary matter. Mr. Harland (the French-American editor who makes his home in England), has two graceful historiettes. They have neither of them the virility of "Mademoiselle Miss" or of "Bibi," but their charm is undeniable. We know some children who already squabble for the Yellowbook as they all want to read "Mercedes" at once. Henry James and Fred. Simpson should not have been included in the same volume so very much at the expense of the latter, as their "motiv" is the same—the disastrous effect that success is apt to have on genius. Henry James treats his subject *en maître*, but the little play, "Dedication," though pretty in conception, is the evident "amateur effort." Ella D'Arcy is a new writer, and her "Irremediable" is very clever indeed. It is seldom that a young woman is able to plumb a young man's soul with such accuracy. The "advanced school" trouble themselves merely to depict their modern woman as contrasted with the conventional woman—the men they draw are not men but just pegs to hang vices upon, which shall provide the "advanced woman's" texts. Miss D'Arcy shows us how a young man may mar his whole life by endeavouring to act most virtuously towards a young woman who is—from a society point of view—"quite impossible." "Modern Melodrama" is, no doubt, a piece of very "up to date" literature, by Hugh Crackanthorpe, the author of "Wreckage," but it seems to us a wholly unnecessary piece of work, neither artistic or beautiful; piteous, perhaps, but unpleasing—as disagreeable to look at as a gangrene. George Egerton (the Mrs. Clairmonte, author of "Keynotes," about whom so many inaccuracies have recently been written), in her "Lost Masterpiece" tells with a very pretty touch how she had to come up to town for a day's shopping, and how all the passing sights of road and river inspired her with the germ of a great idea. As the day wore on the idea grew, and she fancied she was on the high road to producing a masterpiece; the cause of disaster it is not fair to reveal, but the author cleverly wins our sympathy for herself and our detestation of "the woman with the white-handled umbrella." In "The Fool's Hour" J. O. Hobbes and George Moore have built up many of their favourite paradoxes, and have attempted, moreover, the composition of epigrams. Mrs. Craigie did these better when she worked by herself; since she has come under the influence of George Moore her books have lost in general interest. "Emotions and a Moral" and the "Sinner's Comedy" were infinitely superior to the foolish "Bundle of Life," with its affectations of "smartness" allied to "les précieuses'" very own style. But enough has been said to prove that the "Yellowbook," if not quite so startlingly eccentric as was expected, is full of interesting and unconventional matter, which may amuse, and even unwittingly teach, by causing one to consider some of its propositions and to contradict others. There is much that women will say "no" to in Max Beerbohm's "Defence of Cosmetics."

### A Modern Heretic: The Story of a Scismatic. (James Clark and Sons. 1894.)

This is a novel written seriously, for serious-minded readers. It is a novel with a purpose; the purpose being the denouncing of the privileged position usurped by our Established Church. Those not interested in the question of dissent in the English Church would find small pleasure in the pages of "A Modern Heretic," as the interest of the book is confined to this subject. It would have been better, perhaps, had the author bestowed more trouble on the "style" of his writing, on which he honestly admits "no labour will be bestowed."

### MAGAZINES.

This month's Idler opens with a very good story of a lady doctor by Conan Doyle. Miss Duncan's amusing adventures of a maiden aunt visiting her nephew in India are continued. In addition to a very good paper on Japanese habits and customs by D-Gom of the Japanese Legation, there is much that is very interesting in "Henri Rochefort at Home." One is struck by rather a remarkable and unexpected answer to the question addressed to him by his interlocutor, "How do you regard bomb-throwing Anarchism?" "Such things," replies the Marquis, "do incalculable harm to the cause of Liberty, and though you will probably not share my opinion, I may tell you that I am firmly convinced that many so-called Anarchist outrages are really planned and carried out by the police. The same may be said of political assassinations. It is so easy to use as a tool some poor half-crazy fool, who hopes in this way to win notoriety and fame." This certainly puts an entirely new view on this terrible latter-day scourge, and M. Rochefort's experiences as journalist, dramatist, ex-convict, and exile ought to have taught him something. The "Idlers' Club" pleasantly discusses type-writing, which will make clear to the uninitiated the uses and abuses of type-writers. Anyhow, one thing is indisputable regarding them, and that is that they are invaluable to the unintelligible calligrapher, also to the reader of that writer's workmanship.

